# Serial Passaging of RAW 264.7 Cells Modulates Intracellular AGE Formation and Downregulates RANKL-Induced In Vitro Osteoclastogenesis

**DOI:** 10.3390/ijms23042371

**Published:** 2022-02-21

**Authors:** Tanzima Tarannum Lucy, A. N. M. Mamun-Or-Rashid, Masayuki Yagi, Yoshikazu Yonei

**Affiliations:** Anti-Aging Medical Research Center and Glycation Stress Research Center, Graduate School of Life and Medical Sciences, Doshisha University, Kyoto 610-0394, Japan; amamun@mail.doshisha.ac.jp (A.N.M.M.-O.-R.); myagi@mail.doshisha.ac.jp (M.Y.); yyonei@mail.doshisha.ac.jp (Y.Y.)

**Keywords:** serial passaging, RAW 264.7 cells, advanced glycation end products, osteoclastogenesis, glycation, glycative stress

## Abstract

The passage number of cells refers to the number of subculturing processes that the cells have undergone. The effect of passage number on morphological and phenotypical characteristics of cells is of great importance. Advanced glycation end products have also been associated with cell functionality and characteristics. Murine monocyte RAW 264.7 cells differentiate into osteoclasts upon receptor activation caused by nuclear factor-kappa-Β ligand (RANKL) treatment. This study aims to identify the role of passage number on intracellular advanced glycation end products (AGEs) formation and osteoclastogenic differentiation of RAW 264.7 cells. Western blotting was performed to check intracellular AGE formation along with fluorometric analysis using a microplate reader. Tartrate-resistant acid phosphatase (TRAP) staining was performed to check osteoclastogenic differentiation, and qPCR was realized to check the responsible mRNA expression. Immunofluorescence was used to check the morphological changes. Intracellular AGE formation was increased with passaging, and the higher passage number inhibited multinucleated osteoclastogenic differentiation. Osteoclastogenic gene expression also showed a reducing trend in higher passages, along with a significant reduction in F-actin ring size and number. Lower passages should be used to avoid the effects of cell subculturing in in vitro osteoclastogenesis study using RAW 264.7 cells.

## 1. Introduction

To study various cell biology experiments including drug development, drug susceptibility testing, identification of gene function, and cell-cell interaction, numerous in vitro cell culture models have been used for decades [1]. Regular culturing and subculturing of cells are the basic steps for maintaining cell viability in in vitro conditions. The passaging of cells refers to a process starting from cell seeding followed by a certain period of incubation to allow them to grow for multiple generations, then transferring them into a new cell culture flask to allow them to re-grow. The passage number is the degree of subculturing that the cell line has undergone. In other word, In other word, it is the split number of cells [2]. There have been several studies regarding the effects of passage number on the characteristics of the cell population. It has been reported that directly isolated primary cells from living tissues [3] undergo morphological changes and progressive damage with increasing passage number [4]. The reproducibility of data of various in vitro studies may be affected as a result of the passaging process; hence, it can be a source of variation. Moreover, these changes occur at relatively low passage numbers for some cell lines, whereas, at high passage numbers for other cell lines [2]. Several studies with several types of cell lines report that gradual cessation of mitotic activities, increased generation time, and accumulation of cellular debris were observed in cells at high passage numbers [4,5,6,7]. Conversely, Lin et al. report that the LNCaP cell population is over two times higher after five days at passage number 70 than that at passage number 38 [8]. Bonab et al. report that, in long-term cultures, several physiological, functional, and molecular parameter changes occur, including a gradual decrease in proliferation potential, impairment of functions, typical Hayflick phenomenon of cellular aging, and shortening of the telomeres in the MSC cell line [9].

Being monocyte/macrophage-like cells and originating from the Abelson leukemia virus-transformed cell line from BALB/c mice, RAW 264.7 cells are considered an appropriate model of macrophage and are used as an in vitro osteoclastogenesis model for many years. The American Type Culture Collection (ATCC), the main supplier of this cell line, recommends the use of RAW 264.7 cells until passage 18, due to a loss in the ability to differentiate into osteoclast, as per the report from one study [10]. Our laboratory has been using RAW 264.7 cells for a long time, and we also observed a decrease in its differentiation potential with increased passage numbers. Therefore, we always used early passages, passages 3 to 6, in all our in vitro osteoclastogenesis studies [11,12,13,14,15]. However, for other studies, such as in vitro inflammatory cytokine production, we did not notice changes in cell function and reproducibility until passage 20 [16,17,18].

The glycation reaction refers to the reaction between protein or nucleic acid and sugar or sugar derivatives, giving rise to advanced glycation end products (AGEs). AGEs have been associated with many diseases, such as diabetes and its complications [19,20,21,22], Alzheimer’s [23,24], bone diseases [20,23,25,26,27], and chronic kidney disease [26]. AGEs have also been linked with inducing cell cycle arrest [28], apoptosis [29], migration, and proliferation of cells [28,30], which are considered as functionality and phenotypic characteristics of cells, and are reported to be altered with the passaging number [8,31]. AGEs are reported to bind with receptor for AGEs (RAGE) and alter intracellular signaling, some of the AGEs, for example CML-HSA *(N^ε^*-carboxymethyllysine-human serum albumin), can induce inflammatory cytokine production [15,16,17,18]. HSA-AGEs and CML-HSA showed inhibitory effects [12], whereas collagen-AGEs showed stimulatory effects on in vitro osteoclastogenesis [12,13]. Several studies showed high levels of pentosidine and CML in blood serum in osteoporotic patients [20,23,25,26,27,28], providing evidence that they are involved in excessive osteoclastic bone resorption and may cause osteoporosis. However, all the reports show the effects of extracellular AGEs on osteoclastogenesis: there is no significant documentation regarding the relation between intracellular AGE levels in the macrophage cell and its osteoclastogenic differentiation potential.

The present study aims to find out the effect of passaging on intracellular AGE formation and accumulation, and on osteoclastogenic differentiation capacity using the RAW 264.7 cell line.

## 2. Results

### 2.1. Relationship between Passage Number of RAW 264.7 Cells with Intracellular AGE Formation and Accumulation

**Figure 1:**
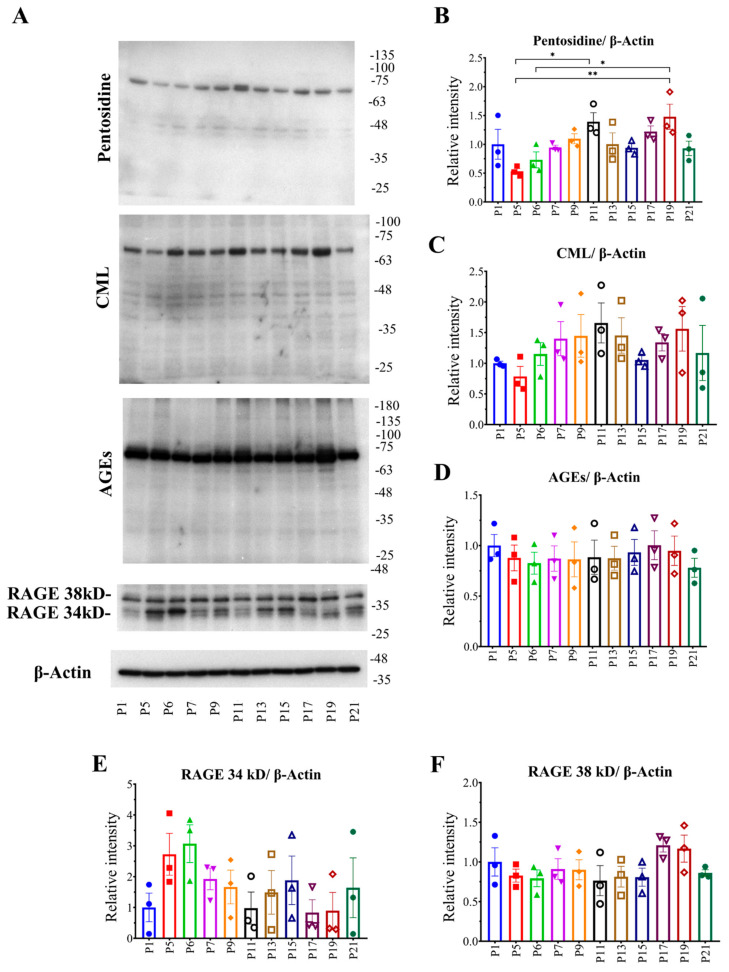
Effect of passaging on intracellular AGE formation. Cells were collected from different passage numbers and cell lysate was prepared. (**A**) Pentosidine, CML, intracellular total AGEs, and RAGE were checked using western blotting. (**B**–**F**) Graphs show ImageJ analysis of band intensity. Values are means ± SEM (*n* = 3, each group), Tukey–Kramer test, * *p* < 0.05, ** *p* < 0.01. RAGE, receptor for AGEs; AGEs, advanced glycation end products; CML, *N*^ε^-carboxymethyllysine; SEM, standard error mean.

Cells from different passages were collected for checking intracellular AGE formation and accumulation. Western blotting was performed to check the intracellular AGEs at the protein level. Intracellular total AGEs, pentosidine, and CML were checked using specific antibodies against them. Pentosidine formation was decreased in passage (P) 5 compared to P1, and then appeared to be increased with passaging P11, and again decreased except for P19. CML formation showed an incrementor trend, except for P5 and P15 compared to P1. The total AGE formation was almost similar in all passages. Receptors for AGEs (RAGE) were also checked. RAGE 34 kDa was maximum at P5 and P6; contrarily, RAGE 38 kDa was almost similar in all passages, except for P17 and P19, where it was highest among all the passages. Collectively, intracellular AGEs (pentosidine and CML) formation and accumulation increased during the subculture process, and cells can either remove them from the cellular body, or the accumulated AGEs are reduced per cell during cell division (Figure 1).

### 2.2. Relationship between Passage Number of RAW264.7 Cells with Intracellular Fluorescent AGE Formation and Accumulation

**Figure 2:**
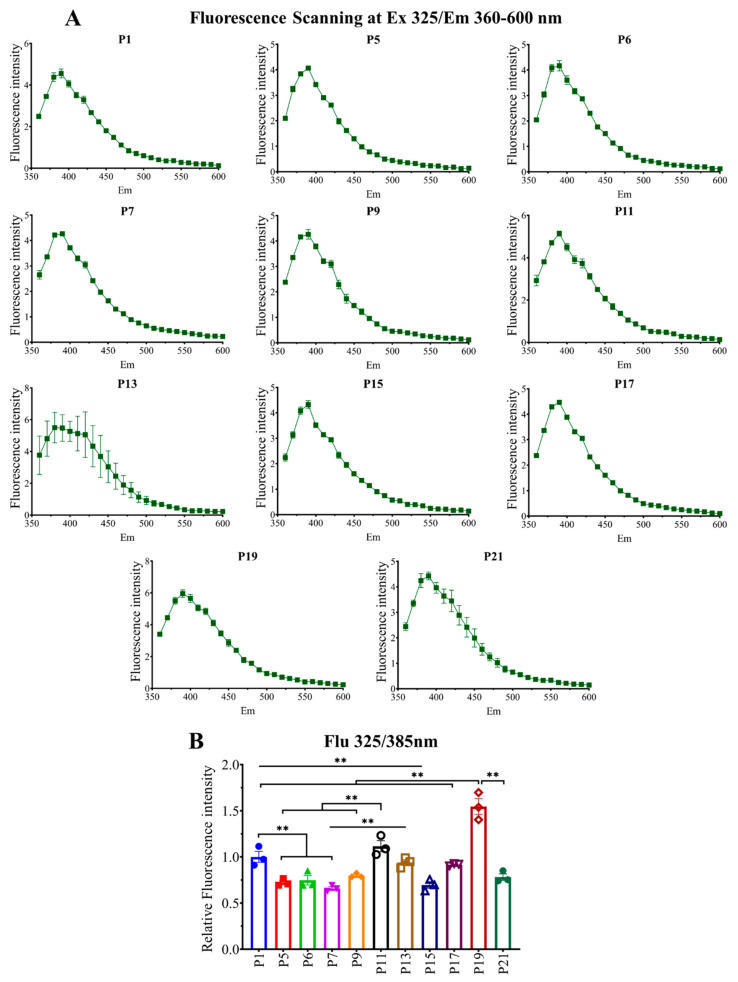
Fluorescent AGEs formation and accumulation at different passages. (**A**) Fluorescence scanning at ex 325/em 360–600 nm. (**B**) Fluorescence intensity at ex 325/em 385 nm. All data are shown as means ± SEM, *n* = 3. ** *p* < 0.01 by the Tukey-Kramer test.

**Figure 3:**
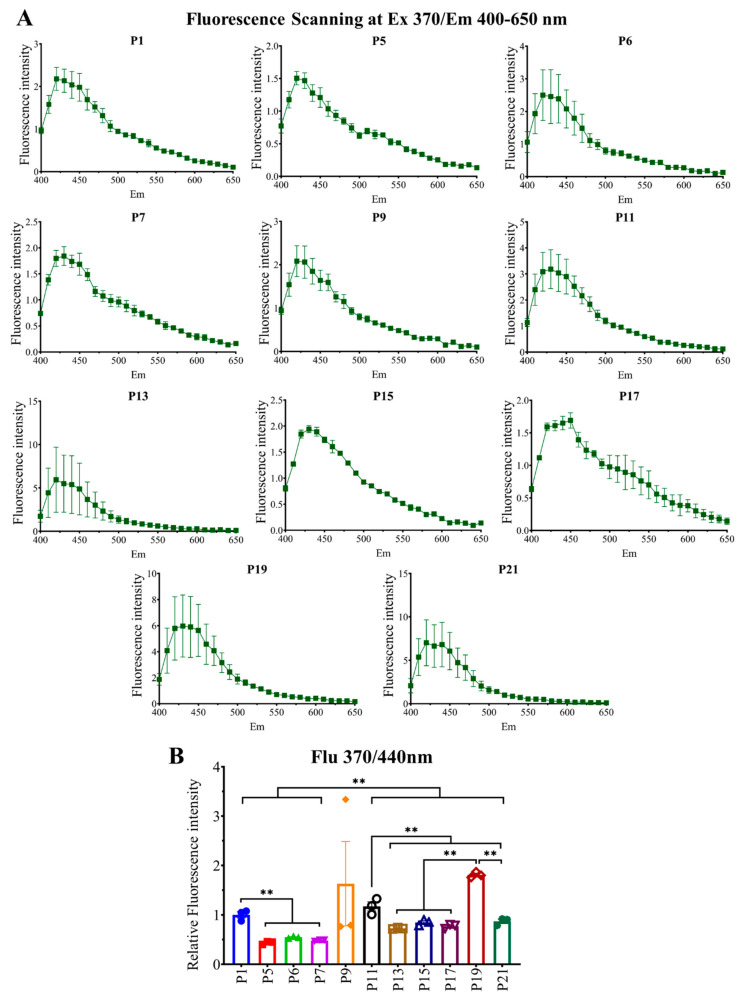
Fluorescent AGEs formation and accumulation at different passages. (**A**) Fluorescence scanning at ex 370/em 400–650 nm. (**B**) Fluorescence intensity at ex 370/em 440 nm. All data are shown as means ± SEM, *n* = 3. ** *p* < 0.01 by the Tukey–Kramer test.

Cells from different passages were collected, and an equal amount of cell lysates was used to check intracellular fluorescent AGE formation and accumulation. Intracellular fluorescent AGEs were checked using a microplate reader. Pentosidine yields fluorescence at excitation/emission (ex/em) 325/385 nm (Figure 2); moreover, some unidentified AGEs showed fluorescence at the same wavelength. Other fluorescent AGEs were collectively measured at ex/em 370/440 nm (Figure 3). We scanned at ex/em 325/360–600 nm (Figure 2A) and 370/400–650 nm (Figure 3A) to see if any other AGEs were being produced which showed fluorescence emission at this range. The formation and accumulation of fluorescent AGEs (ex/em 325/385) was significantly reduced upon culturing from P1 to P5–P7, then showed a significant increase in P11–P13, followed by a fall in P15–17 and a rise in P19 again; later, in P21 it decreased significantly (Figure 2B). Fluorescent AGEs (ex/em 370/440 nm) were reduced in early passages P5–P7 but showed a significant increase in later passage P11–P21 (Figure 3B). These data show that there is a significant number of AGEs being produced and accumulated during cell culture, and cells are capable of excreting them from intracellular space. That is why the intracellular fluorescent AGE level shows up and down curves.

### 2.3. Increasing Passage Number Significantly Inhibited Osteoclastogenic Differentiation

**Figure 4:**
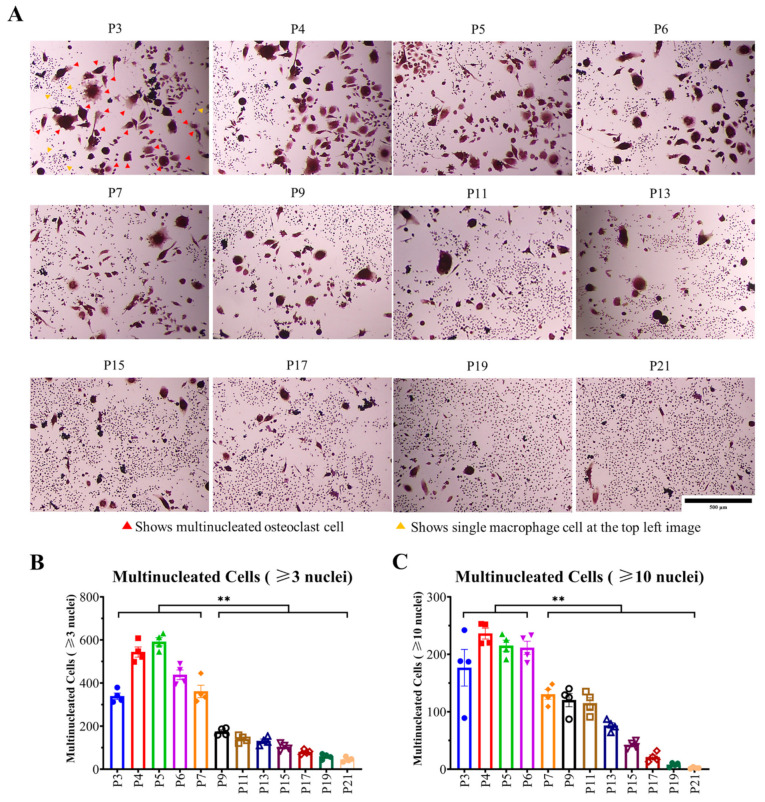
Effect of passage number on multinucleated TRAP-positive cell formation. RAW 264.7 cells were plated in 96-well plates at 1 × 10^4^ cells/well and incubated for 24 h followed by treating with αMEM containing 10% FBS, 100 ng/mL RANKL. After three days, the media was renewed and after five days, cells were used for TRAP staining. (**A**) multinucleated TRAP-positive cells, (**B**) multinucleated cells (≥3 nuclei), and (**C**) multinucleated cells (≥10 nuclei). All data are shown as means ± SEM, *n* = 4. ** *p* < 0.01, Tukey–Kramer test. The bar in the figure represents 500 µm, the yellow triangle indicates single macrophage cells, and the red triangle indicates multinucleated osteoclast cells at the top left image.

To determine the effect of sub-culturing of cells over multiple times on osteoclastogenic differentiation, TRAP staining (Figure 4A) was performed after treating the cells with 100 ng/mL RANKL. Cells with (≥3 nuclei) and cells with (≥10 nuclei) were counted as multinucleated osteoclast. Multinucleated osteoclast (≥3 nuclei) formation was higher from P3 to P7, which was significantly declined from P9 (Figure 4B). On the other hand, the formation of multinucleated giant osteoclast (≥10 nuclei) was higher from P3 to P6, which was significantly declined from P7 (Figure 4C). These data reveal that increasing passage number inhibits multinucleated osteoclast formation.

### 2.4. Higher Passage Number Reduced F-Actin Ring Size Significantly

**Figure 5:**
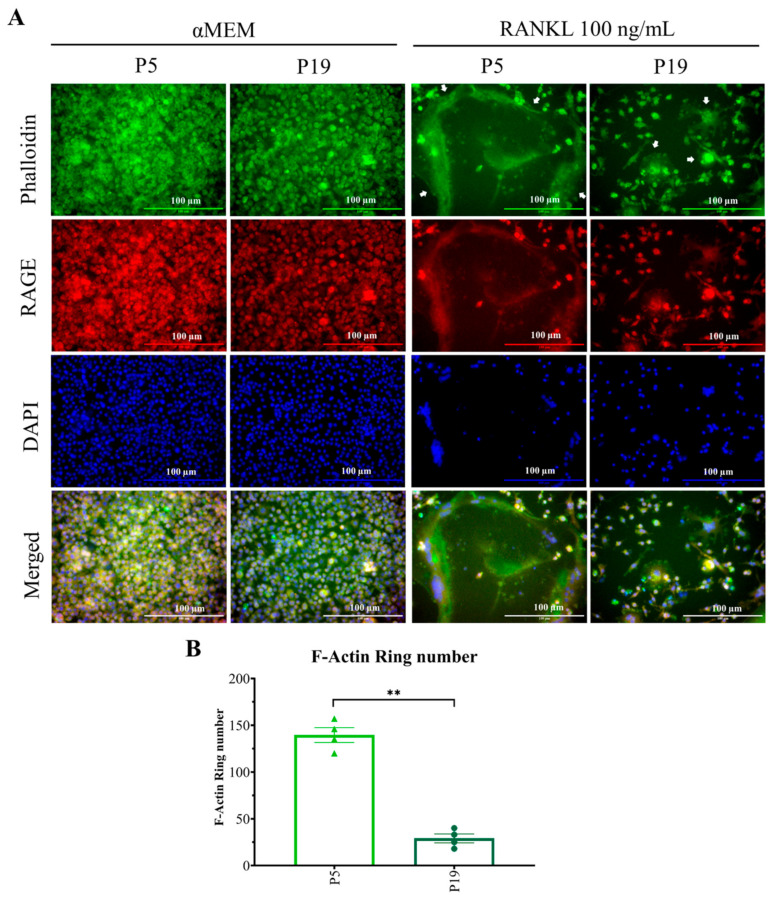
Effect of passage number on F-actin ring formation. (**A**) Cells were seeded and treated same as the TRAP staining experiment followed by staining with phalloidin to stain the cytoplasm (green), an antibody against RAGE (red), and DAPI (blue) to stain the nucleus. The bar in the figure represents 100 μm. White arrows indicate osteoclast cells. (**B**) F-actin ring number in two different passages. ** *p* < 0.01, Tukey–Kramer test.

The F-actin ring is a special characteristic feature of osteoclast cells, which indicates and is formed by cell-cell fusion [15,32]. The effect of passage number on F-actin ring size was checked upon 100 ng/mL RANKL stimulation by using immunofluorescence staining. Cells from two passages were taken: P5 and P19. The size of the F-actin ring in P19 was significantly reduced compared to P5, indicating possible evidence for decreasing cell fusion in higher number of passages (Figure 5A). The numbers of F-actin rings were also significantly reduced in higher passages, providing evidence of reducing cell–cell fusion between cells of higher passage. Our study clearly showed that the number of osteoclasts was significantly reduced, and the osteoclast size was very small in higher passages due to reduced cell-cell fusion (Figure 4 and Figure 5).

### 2.5. Higher Passage Number Reduced Osteoclastogenic Gene Expression

**Figure 6:**
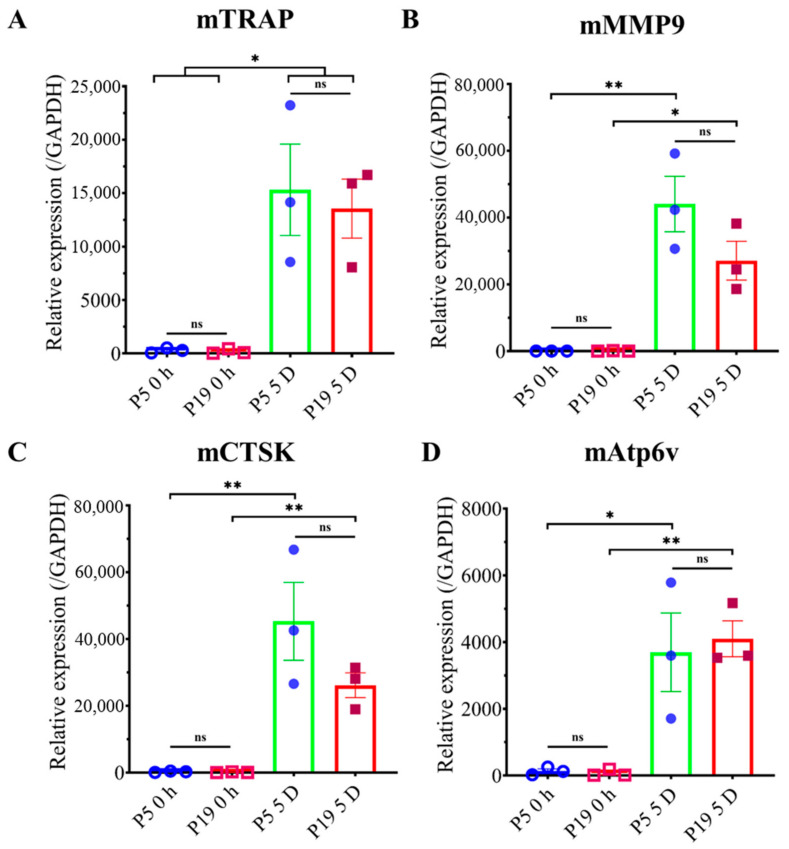
Effect of passage number on maturation marker gene expression. RAW 264.7 cells from two different passages were seeded in 24-well plates at 4 × 10^4^ cells/well. After 24 h, the media were changed with ⍺MEM containing 10% FBS without or with 100 ng/mL RANKL. After three days, the medium was renewed. After five days of total treatment, the treated cells were collected, and mRNA was extracted. Then, these were used for cDNA synthesis and checked by qPCR to verify the mRNA expression of (**A**) TRAP, (**B**) MMP 9, (**C**) CTSK, and (**D**) Atp6v. Relative mRNA expression, data were normalized by GAPDH. All data are shown as means ± SEM, *n* = 3. * *p* < 0.05, ** *p* < 0.01, ns—non significant. Tukey–Kramer test.

**Figure 7:**
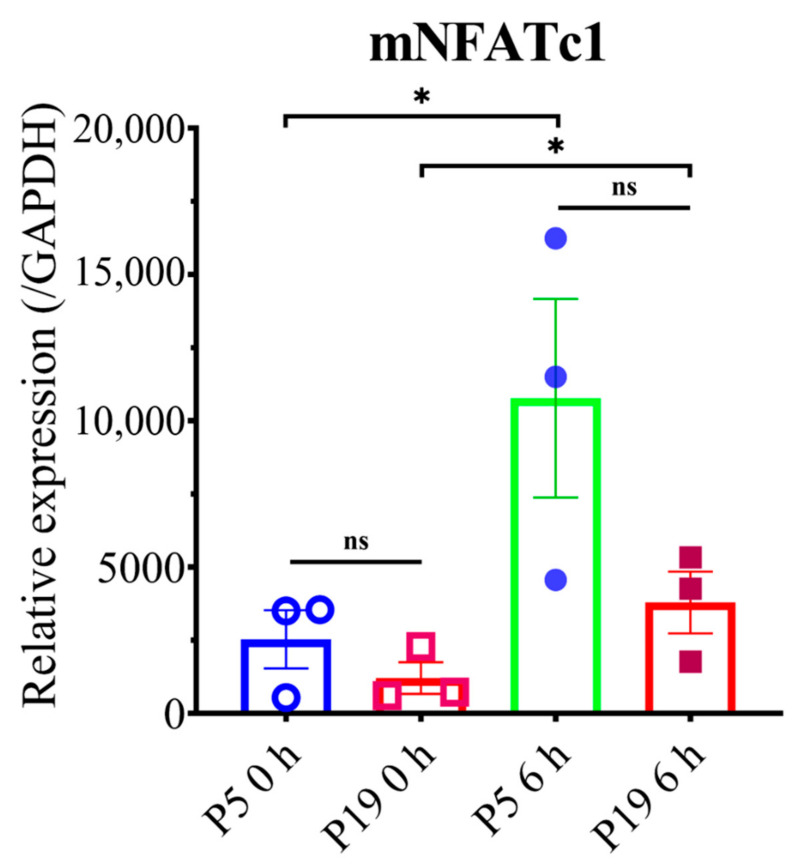
Effect of passage number on early marker gene expression. RAW 264.7 cells from two different passages were plated in 24-well plates at 4 × 10^4^ cells/well. The next day, cells were treated with ⍺MEM containing 10% FBS without or with 100 ng/mL RANKL. After 6 h of treatment, the cells were collected, mRNA was extracted, cDNA synthesis was performed, and mRNA expression for NFATc1 was checked by qPCR. Relative mRNA expression, data were normalized by GAPDH. All data are shown as means ± SEM, *n* = 3. * *p* < 0.05, ns—non significant. Tukey–Kramer test.

Cells from P5 and P19 were used to check mRNA expression for maturation and early osteoclastogenic markers. In the case of expression of the maturation marker gene, cells were seeded and treated without or with RANKL, and after five days of treatment, mTRAP, mAtp6v0, mMMP9, and mCTSK were checked. All the mRNA expressions, except for mAtp6v0, showed an almost similar trend, which is slightly decreased in P19 than P5 (Figure 6). The early maturation marker was checked after 6 h of treatment without or with RANKL; mNFATc1 expression was slightly low by P19 compared to P5 (Figure 7). These data show that mRNA expression was not significantly changed even though the ultimate osteoclastogenesis was significantly reduced.

### 2.6. Correlation Study

**Figure 8:**
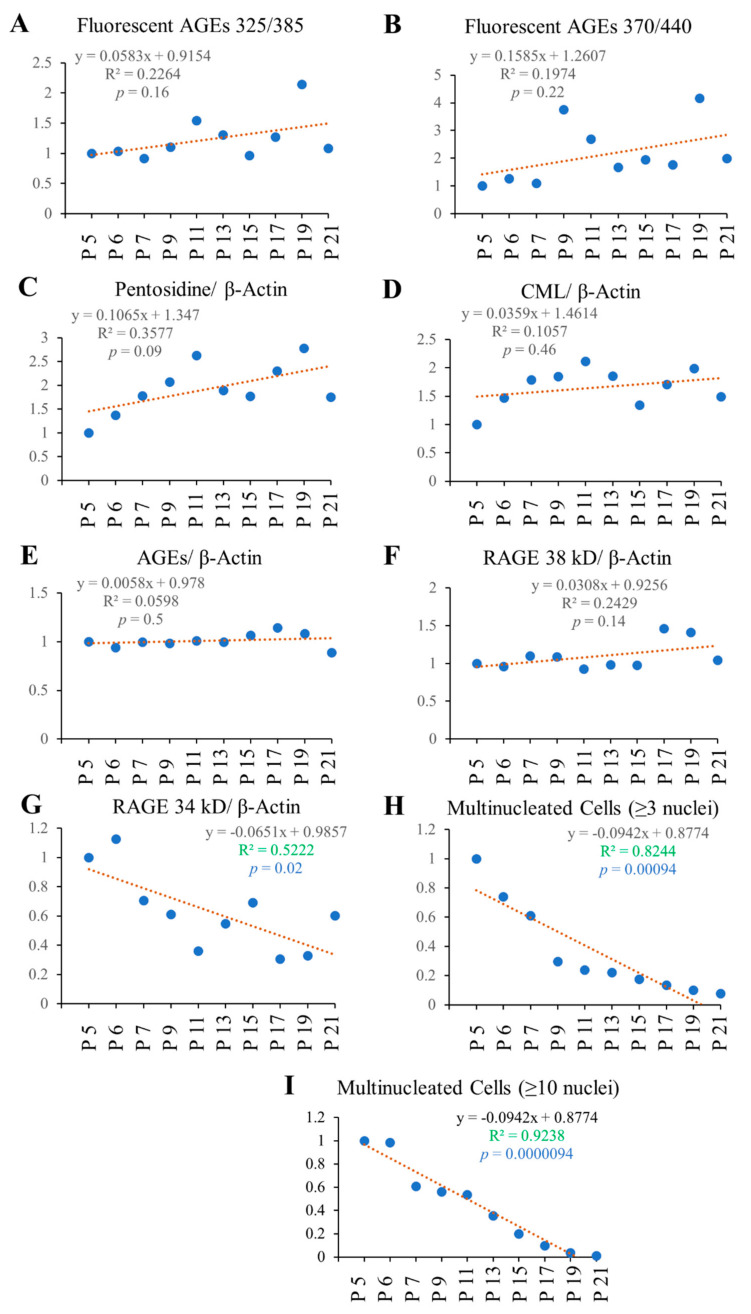
Correlation analysis of passage number with different features as mentioned in the figures. Higher R^2^ and lower *p*-value (<0.05) indicate highly correlated statistically significant data. Correlation of (**A**) Fluorescent AGEs 325/385, (**B**) Fluorescent AGEs 370/440, (**C**) Pentosidine/β-actin, (**D**) CML/β-actin, (**E**) AGEs/β-actin, (**F**) RAGE 38 kD/β-actin, (**G**) RAGE 34 kD/β-actin, (**H**) Multinucleated cells (≥3 nuclei), and (**I**) Multinucleated cells (≥10 nuclei) with passage numbers.

**Figure 9:**
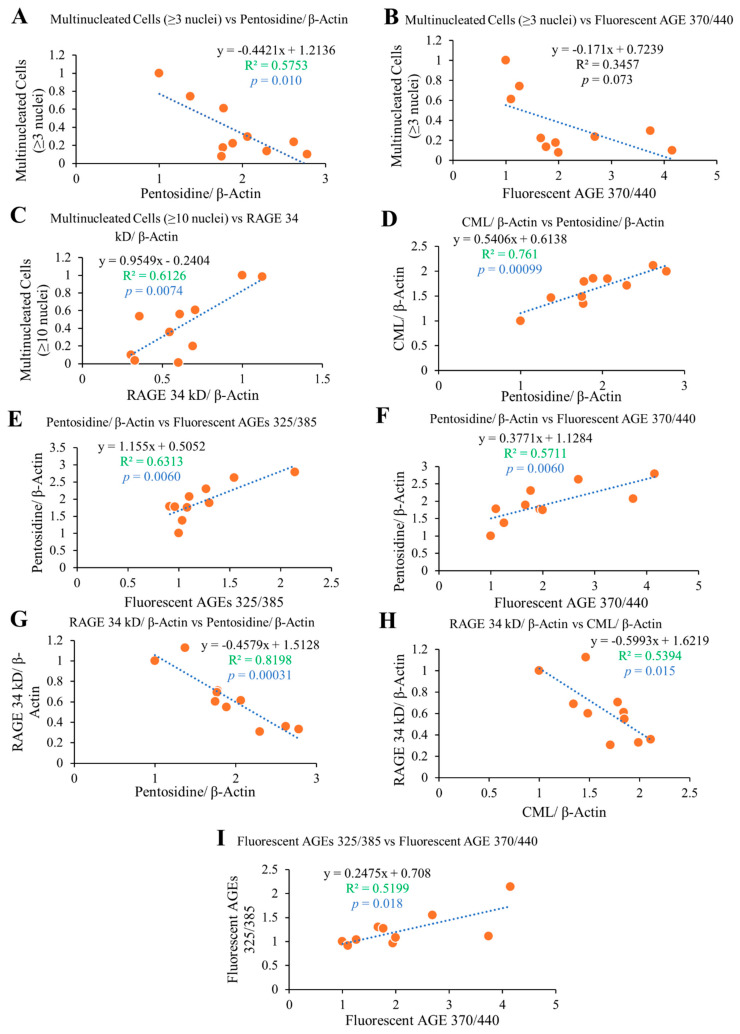
Correlation analysis of different features as mentioned in the figures. Higher R^2^ and lower *p*-value (<0.05) indicate highly correlated statistically significant data. (**A**) Multinucleated cells (≥3 nuclei) vs. Pentosidine/β-actin, (**B**) Multinucleated cells (≥3 nuclei) vs. Fluorescent AGE 370/440, (**C**) Multinucleated cells (≥10 nuclei) vs. RAGE 34 kD/β-actin, (**D**) CML/β-actin vs. Pentosidine/β-actin, (**E**) Pentosidine/β-actin vs. Fluorescent AGEs 325/385, (**F**) Pentosidine/β-actin vs. Fluorescent AGE 370/440, (**G**) RAGE 34 kD/β-actin vs. Pentosidine/β-actin, (**H**) RAGE 34 kD/β-actin vs. CML/β-actin, and (**I**) Fluorescent AGEs 325/385 vs. Fluorescent AGE 370/440.

The correlation study reveals that fluorescent AGEs and pentosidine formation showed an increasing trend with passage number, while RAGE 34 kD and multinucleated cell formation (both having ≥3 or 10 nuclei) showed a significant decreasing trend with passage number (Figure 8). Multinucleated (≥3 nuclei) cell formation showed a high inverse correlation with intracellular pentosidine (*p* = 0.010) and fluorescent AGE 370/440 (*p* = 0.073), providing evidence for reduced osteoclast formation upon increased intracellular pentosidine and fluorescent AGE 370/440 formation (Figure 9A,B). Pentosidine and fluorescent AGE 370/440 formation may cause structural and functional loss of the proteins that are responsible for cell–cell fusion, and thereby reduced osteoclastogenesis. Multinucleated giant (≥10 nuclei) cell formation showed a high positive correlation with RAGE 34 kD expression (*p* = 0.0074), providing evidence for the involvement of RAGE 34 kD on the cell–cell fusion and osteoclastogenic differentiation, as higher expression of RAGE attributed with the higher number of giant osteoclast cell formation (Figure 9C). With the reduced expression of RAGE 34 kD, multinucleated osteoclast formation was also reduced with cell passaging. RAGE was reported to play vital role on cell–cell fusion by binding with extracellular HMGB1 [33], and thereby induce osteoclastogenesis and its function. RAGE-deficient osteoclasts showed disrupted F-actin ring formation as well as sealing zones due to impaired maturation, and reduced bone resorption in in in vitro differentiation condition [34]. Reduction of RAGE expression could be another reason of reduced osteoclastogenesis in our study.

Intracellular pentosidine formation was positively correlated with intracellular CML (*p* = 0.00099; Figure 9D), fluorescent AGEs 325/385 (*p* = 0.0060; Figure 9E), and fluorescent AGEs 370/440 (*p* = 0.0060; Figure 9F) formation, providing evidence for increased intracellular AGE formation and accumulation attributed to all types of AGEs instead of any one kind of AGE. Intracellular pentosidine (*p* = 0.00031; Figure 9G) and CML (*p* = 0.015; Figure 9H) formation was negatively correlated with RAGE 34 kD expression, providing evidence for higher intracellular AGE formation may cause reduced RAGE 34 kD expression. Fluorescent AGEs 325/385 formation showed a higher positive correlation with fluorescent AGEs 370/440 (*p* = 0.018; Figure 9I). Collectively, these data show that increased level of intracellular AGE formation attributed to the reduced level of RAGE 34 kD expression, and at the same time reduced osteoclastogenic differentiation. RAGE has been reported to play a vital role in osteoclastogenesis, and any downregulation of RAGE showed a significant reduction in osteoclastogenesis [12,33,34]; our current study also provides similar information.

**Figure 10:**
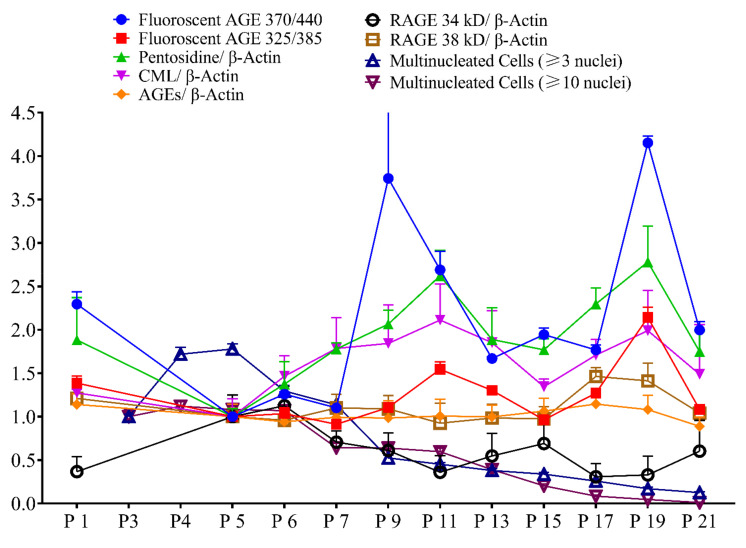
The trend of different features together with passage number.

Multinucleated cell number and RAGE 34 kDa showed a gradual decline with passage number, while fluorescent AGE, pentosidine, CML, and RAGE 38 kDa showed a gradual increase with passage number (Figure 10).

## 3. Discussion

RAW 264.7 cells have gained popularity as an appropriate macrophage model for in vitro study over bone marrow macrophage (BMM) cells due to their easy culturing and passaging techniques, homogeneity, extensive availability of the cell line, and close correlation in gene expression, characteristics, signaling, and functional or developmental processes among the primary precursor cell linage derived osteoclast. RAW 264.7 cell differentiated into the osteoclast, and isolated in vivo formed osteoclasts [10]. Passaging is an important part of this cell culture study, and the influence of the passaging number on data reproducibility cannot be ignored. There have been several studies revealing the effects of passage number on cell phenotypes, gene expressions, metabolomics, and functions using MCF7 [35], U87 [36], OEC [36], MC3T3-E1 [37], PC12 [38], HUVEC [39], D1 [40], RAW 264.7 [41], and HT29 cells [36]. However, there has been no study regarding the effect of passage number on intracellular AGE formation and osteoclastogenic differentiation. 

Since AGEs formed in cells are also responsible for some phenotypic changes in cells, we hypothesized that there could be some effects of passaging on the formation of intracellular AGEs. Subculturing of cells was performed until passage number 21, and, using the cell lysate, Western blot analysis was conducted to check pentosidine, CML, AGEs, and receptor for AGEs (RAGE). Pentosidine and CML are well-known AGEs, as well as biomarkers for type 2 diabetic retinopathy [19,21], age-related macular degeneration [22], and are associated with the prevalence of osteoporotic bone fracture and inflammation [20,21,22,23,25,26,27,28,29]. RAGE plays an important role by binding with AGEs in some diseases [12,24,28,33]. From our data, we found that passaging significantly affects intracellular AGE formation, including pentosidine, CML, fluorescent AGEs, and cells can reduce intracellular AGEs either by excreting them or during cell division, but there is a significant reduction in intracellular RAGE expression (Figure 8G). The data indicate that the cells are continuously facing glycative stress, thereby facing increased levels of cellular AGEs, and cells attempt to excrete the intracellular AGEs in order to retain the structure and function of the cellular proteins.

Later, we checked fluorescent AGE formation ex/em at 325/385 nm and 370/440 nm. Pentosidine and some other unidentified AGEs yield fluorescence at ex/em 325/385 nm, and, in Figure 2, we found that it was affected by the serial passaging. In the case of fluorescent AGEs derived fluorescence intensity at ex/em 370/440 nm, a higher passage number (P9–P21) yielded higher fluorescence intensity compared to lower passage (P1–P7), which suggests the possibility of producing higher fluorescent AGEs in cells with higher passage number (Figure 3).

Afterward, to elucidate the effect of passage number on RAW 264.7 cell differentiation into osteoclast, TRAP staining was performed, which revealed that multinucleated cell formation was significantly inhibited with increasing passage number; from P3–P7, osteoclastogenic differentiation was the highest, which started to decrease significantly afterward (Figure 4). The above-mentioned data (Figure 3 and Figure 4) indicate that there might be an association between fluorescent AGEs and osteoclastogenic differentiation with passage number of RAW 264.7 cells.

To evaluate the mechanism underlying decreasing osteoclast differentiation, P5 and P19 were chosen to perform F-actin ring formation through immunofluorescence. F-actin ring is the actin cytoskeletal reorganization and is required for giant osteoclast formation and activation. The ring size also indicates the fusion status of osteoclasts [32]. F-actin ring formation was significantly inhibited in P19, compared to P5, which clearly suggests that multinucleated osteoclast formation, as well as the cell activity decreases with passage number (Figure 4 and Figure 5). Later, the osteoclast maturation marker and early marker were checked. In both cases, after 0-h incubation, gene expression was almost nil, but after the mentioned time of incubation, P5 showed comparatively higher expression of all genes with the exception of mAtp6v compared to P19. Due to a high standard error of mean, the data did not show any significance, but there is a trend of higher maturation marker gene (mTRAP, mMMP9, and mCTSK) and the early marker gene (mNFATc1) expression in lower passage cells compared to higher passage cells which complement our decreased osteoclastogenic differentiation with increasing passage number.

RAW 264.7 cells were investigated from passage 5 to passage 50, and reported that the phenotypic (determined by some of the macrophage-specific genes and surface markers expression) and functional stability (determined by phagocytosis assay and NO production assay) remained stable from passage 10 to passage 30 in a recent study [42]. However, they did not investigate osteoclastogenesis. Another study reported that RAW 264.7 cells differentiate into osteoclast from passage 4, and after 18–20 passages, they completely lost their differentiation potential (we also observed similar phenomena) for an unknown reason. Here, we are reporting for the first time that intracellular AGE formation and accumulation significantly reduce RAGE 34 kD expression and inhibit osteoclastogenic differentiation in an in vitro model. This study could attract further investigation on how intracellular AGEs affect cell–cell fusion and differentiation, in which proteins are glycated quickly, and how to prevent intracellular glycative stress. Moreover, there could also be other reasons. In our previous study, we have reported that RAW 264.7 cells undergo proliferation first after RANKL-treatment, and then the new cells participate in cell–cell fusion and differentiation [14]. Any fall in proliferation potential, typical Hayflick phenomenon of cellular aging, impairment of cellular functions, or telomere shortening may also affect the osteoclastogenesis.

We have evaluated the effect of passaging on osteoclastogenesis by counting multinucleated TRAP-positive cell numbers, F-actin ring size and number, and osteoclastogenic gene expression by qPCR. However, there were some limitations in this study. We did not conduct a bone resorption assay. As our data of multinucleated cell size (Figure 4A) and number (Figure 4B), along with F-actin ring size (Figure 5A) and number (Figure 5B), clearly indicate that osteoclastogenesis, as well as osteoclast size, are significantly reduced in higher passage numbers, we therefore believe that bone resorption will not be similar or to the same extent by the small sized osteoclast of higher passage compared to larger sized osteoclasts of lower passage. Another limitation was that we could not detect the excretion of intracellular AGEs into culture media due to high fluorescence and unspecific antibody binding with FBS.

The purpose of the present study was to investigate the effect of serial passaging on intracellular AGE formation and osteoclastogenesis. This study shows some evidence of intracellular AGE formation and accumulation, as well as removal under in vitro culture conditions and a dramatic fall of osteoclastogenesis after a few passaging cycles.

## 4. Materials and Methods

### 4.1. Cell Culture

Murine monocyte/macrophage RAW 264.7 (ATCC TIB-71) cell was bought from American Type Culture Collection (ATCC; Manassas, VA, USA). For the culturing of cells, Dulbecco’s modified Eagle’s medium (DMEM; Sigma-Aldrich, St. Louis, MO, USA) with high glucose was used along with 10% fetal bovine serum (FBS) (Nichirei Biosciences, Tokyo, Japan) to maintain the nutrition of the media. Penicillin 100 units/mL, streptomycin 100 µg/mL, and amphotericin B 25 µg/mL (Gibco, El Paso, TX, USA) were added to the media as antibiotics. Cells were incubated at 37 °C temperature and 5% CO_2_ [17]. After receiving (third passage), RAW264.7 cells were thawed, centrifuged to remove cryopreservatives, and placed in 10 cm culture dish along with culture media containing FBS and antibiotics. After the cell reach 80% confluency, the cells were scraped and subcultured in new dish at 5 × 10^5^ cells/dish and labeled as passage-1 (P-1), and these subculturing was continued until P-4 to get sufficient numbers of cells. Then, several cryovials (of P-4 cells) were prepared for preservation at the liquid nitrogen vapor phase as per manufacturer’s recommendation. These cryovials were then used for all the studies, where thawed cells were labeled as P-0. Each scraping and subculturing was counted as serial passaging, and labeled as P-1 to P-21. We used same parental cells (cryovial) for the same kind of studies to compare between lower passage and higher passage cells.

### 4.2. TRAP Staining

TRAP staining was performed as per the manufacturer’s protocol. Briefly, cells of different passages were seeded at a concentration of 1 × 10^4^ cells/mL in 96-well transparent plate and incubated for 24 h, followed by changing of the media with αMEM (Gibco) with or without 100 ng/mL recombinant mouse RANKL (rmRANKL, R&D Systems, Minneapolis, MN, USA) for five days. Media was renewed after three days. Cells were fixed with 10% formalin neutral buffer solution after culture for a total of five days with differentiation medium (RANKL containing medium). Staining of the cells was completed using a TRAP staining kit (387A-1KT, Sigma-Aldrich, St. Louis, MO, USA). A light microscope (Olympus CKX 41, Tokyo, Japan) was used to count the nuclei and cells. Multinucleated cells having ≥3 nuclei were regarded as osteoclast cells [11,12,13,14,15].

### 4.3. Isolation of Total RNA and q-PCR

Cells were seeded at 1 × 10^5^ cells/well concentration in 24-well plates. Then, 24 h later, cells were treated with or without 100 ng/mL RANKL. In the case of checking the maturation marker gene, after three days, media was renewed, and after a total culture of five days in differentiation media, RNA were extracted. For the early marker gene, 6 h after the first changing of media, RNA was extracted. For total RNA extraction, Isogen II reagent (Nippon Gene, Toyama, Japan) was used according to the manufacturer’s protocol. PrimeScriptTM RT Master Mix (Takara Bio Inc., Shiga, Japan), along with 500 ng of RNase-free Dnase-treated total RNA, was used for reverse transcription by Applied Biosystems 2720 Thermal Cycler. For performing q-PCR, ThunderbirdTM SYBR qPCR mix (Toyobo Co., Ltd., Osaka, Japan) was used, and the gene-specific primers are listed in Table 1 [12,13,15]. The manufacturer’s protocol was followed for the qPCR technique. Briefly, the AB Applied Biosystems StepOnePlus real-time PCR system was used for amplification reactions. An initial hold step (95 °C for 1 min) and 40 cycles of PCR (95 °C for 15 s, 60 °C for 60 s) were carried out, followed by a dissociation curve. To determine the amount of target gene, the comparative CT method was used. For normalization of mRNA expression, GAPDH: glyceraldehyde-3-phosphate dehydrogenase was used.

### 4.4. Protein Extraction and Western Blot Analysis

Cells from different passages were seeded at a concentration of 2 × 10^5^ cells/well in 6-well plate. RIPA buffer containing 50 mM Tris-HCl, 150 mM NaCl, 0.1% SDS, 1% Triton X-100 with complete protease inhibitor (Wako Pure Chemical Industries, Osaka, Japan) was used to lyse the cells. A BCA assay was performed using Pierce BCA Protein Assay Kit, 23225, (Thermo Fisher Scientific, Rockford, IL, USA) to measure the concentration of cell lysate. Electrophoresis of the cell lysates was completed using sodium dodecyl sulfate polyacrylamide gel electrophoresis (SDS-PAGE) (12% polyacrylamide). A polyvinylidene difluoride (PVDF) membrane was used to transfer the protein to the surface, and 5% skim milk solution in TBS-T was used as blocking buffer. Immunoblotting of the membranes with primary antibody was performed using anti-pentosidine (#KH012, Medicinal Chemistry Pharmaceutical, Co., Ltd., Hokkaido, Japan, 1:1000, o/n), anti-CML (#MAB3247, R&D systems, 1:1000, o/n), anti-AGEs (#ab23722, abcam, Cambridge, UK, 1:1000, o/n), anti-RAGE (#ab37647, abcam, 1:5000, o/n), and anti-β-actin (#A5441, Sigma-Aldrich, St. Louis, MO, USA, 1:5000, 1 h). Membranes were washed, and a goat anti-rabbit IgG-HRP secondary antibody (#sc-2004, Santa Cruz Biotechnology, Dallas, TX, 1:10,000, 1 h) or goat anti-mouse IgG-HRP secondary antibody (#7076P2, Cell signaling technology, Danvers, MA, USA, 1:10,000, 1 h) in blocking buffer was used, and after the washing steps, chemiluminescence horseradish peroxidase (HRP) substrate was used to visualize the antigen–antibody complexes along with detection system, as per the manufacturer’s recommendation. The results illustrated in each figure are representative of three independent experiments. The optical density of the protein bands was measured using ImageJ. β-actin was used to normalize the data.

### 4.5. Fluorescence Scanning and Measurement

Cell seeding was performed at a concentration of 2 × 10^5^ cells/well in 6-well plate. Cell lysis was performed using RIPA buffer containing 50 mM Tris-HCl, 150 mM NaCl, 0.1% SDS, and 1% Triton X-100 with complete protease inhibitor (Wako Pure Chemical Industries, Osaka, Japan). A BCA assay was performed to measure the concentration of cell lysate, and 100 µL of 80 μg/mL cell lysate/well was placed on a 96-well black plate, and the fluorescent intensity was measured at ex-em 325/385 nm and 370–440 nm. For fluorescence scanning, excitation was performed at 325 and 370 nm, and the emission was measured from 360–600 and 400–650 nm, respectively. In both cases, a Varioscan Flash fluorometric microplate reader (Thermo Fisher Scientific, Waltham, MA, USA) was used.

### 4.6. Immunofluorescence Assay

Cells of different passages were seeded at a concentration of 1 × 10^4^ cells/mL in 96-well black wall/clear bottom plate and incubated for 24 h followed by changing of the media with or without 100 ng/mL RANKL for five days. Media was renewed after three days. After five days of culture in αMEM or differentiation medium (RANKL-containing media), cells were fixed with 4% formaldehyde at room temperature for 15 min. The cells were blocked with 3%BSA in PBST for 1 h. Thereafter, cells were incubated with the primary antibody in blocking buffer against RAGE ((#ab37647, abcam, 1:1000) at 4 °C overnight. The next day, cells were washed with PBST 3 times. A donkey anti-rabbit IgG secondary antibody conjugated with PE (sc-3745, Santa Cruz Biotechnology, Inc., Dallas, Texas) and Phalloidin-iFlour 488 reagent (#ab176753, abcam, 1:1000) in blocking buffer was used, and incubated at room temperature in the dark for 2 h and washed three times with PBST. Later, cells were incubated with DAPI (PureBlu #135-1303, Bio-rad Laboratories, Berkeley, CA, USA, 1:1000) in milliQ and kept in the dark for 20 min. Lastly, cells were washed three times with milliQ. A fluorescence microscope (Olympus IX71, Tokyo, Japan) was used to manually count F-actin rings and capture representative images. ImageJ [43,44] was used to process the images.

### 4.7. Statistical Analysis

Data were expressed as mean ± standard error of mean (SEM) using GraphPad Prism 8 (GraphPad Software, Inc., San Diego, CA, USA). All statistical analyses were performed using the Tukey–Kramer test for intergroup comparison in all experiments. Differences were considered significant at a significance level of 5%.

## 5. Conclusions

The passage number should be considered during the osteoclast differentiation study using RAW 264.7 murine macrophage cells and according to our study, we suggest using P3-P6 cells to obtain reproducible and reliable data with minimal effect of cell passaging.

## Figures and Tables

**Table 1:** Primer sequences.

Primer Name	Forward	Reverse
NFATc1	GGA GCG GAG AAA CTT TGC G	GTG ACA CTA GGG GAC ACA TAA CT
TRAP	GCG ACC ATT GTT AGC CAC ATA CG	CGT TGA TGT CGC ACA GAG GGA T
CTSK	GAA GAA GAC TCA CCA GAA GCA G	TCC AGG TTA TGG GCA GAG ATT
Atp6v0	ACG GTG ATG TCA CAG CAG ACG T	CCT CTG GAT AGA GCC TGC CGC A
GAPDH	AGG TCG GTG TGA ACG GAT TTG	TGT AGA CCA TGT AGT TGA GGT CA
MMP9	CTG GAC AGC CAG ACA CTA AAG	CTC GCG GCA AGT CTT CAG AG

## Data Availability

The data presented in this study are available on request from the corresponding author.
